# Biopolymeric Films from the White Rind of Watermelon (*Citrullus lanatus*) for Localized 5‑Fluorouracil Application

**DOI:** 10.1021/acsomega.5c06925

**Published:** 2025-09-19

**Authors:** Igor Henrique Cerqueira, Nicole Pichirilli Catirse, Paula de Abreu Fernandes, Saulo Duarte Ozelin, Diógenes dos Santos Dias, Clóvis Augusto Ribeiro, Hernane da Silva Barud, Flávia Aparecida Resende

**Affiliations:** † Department of Biological Sciences and Health, 74374 University of Araraquara (UNIARA), 14801-340 Araraquara, São Paulo, Brazil; ‡ BioSmart Nanotechnology, 14808-162 Araraquara, São Paulo, Brazil; § Institute of Chemistry, São Paulo State University (UNESP), 14800-060 Araraquara, São Paulo, Brazil

## Abstract

In this study, we developed biopolymeric films derived from watermelon white rind (WF) as a platform for the localized delivery of 5-fluorouracil (5-FU). The films were obtained by solvent casting and subjected to comprehensive physicochemical characterization, confirming the successful incorporation of 5-FU and the biocompatibility of the control WF films. Biological assays were performed using murine melanoma (B16–F10) and human keratinocyte (HaCaT) cell lines. WF-5FU films significantly reduced the cell viability, clonogenic survival, and migration in both models. The cytotoxic effects were more pronounced in B16–F10 cells, confirming their higher sensitivity to the treatment. The micronucleus (MN) assay revealed a concentration-dependent increase in mutagenicity, consistent with the known genotoxic profile of 5-FU. Control films did not induce cytotoxic or genotoxic effects. Overall, WF-based biopolymeric films demonstrated favorable safety in the absence of drug loading and exhibited antitumor activity when loaded with 5-FU. These findings support their potential as a sustainable and effective topical delivery system for localized skin cancer therapy. Further in vivo and clinical investigations are needed.

## Graphical abstract

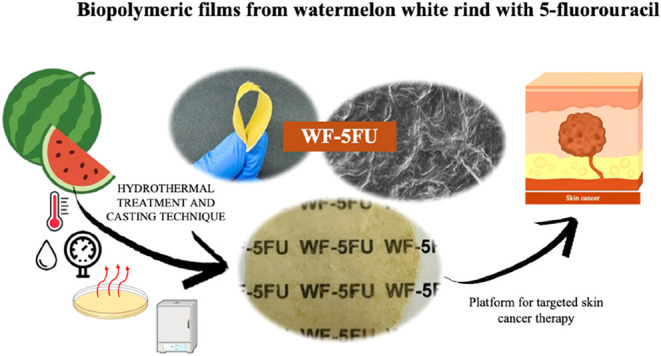

## 1 Introduction

The incidence of skin cancer has been rising significantly, posing a major public health challenge due to increasing mortality rates and treatment costs.^1^ Melanoma is the most aggressive type of skin cancer, characterized by high metastatic potential and lethality.^2^

Therapeutic approaches depend on the stage of the disease and may include surgery, radiotherapy, immunotherapy, chemotherapy, and targeted therapies.^3,4^ However, despite advances in treatment, significant challenges remain, such as tumor resistance and severe side effects, which can compromise both therapeutic efficacy and patient quality of life.^5^ Therefore, the development of innovative drug delivery systems is crucial to enhancing treatment effectiveness while minimizing systemic toxicity.

Polymeric films have emerged as a promising platform for the localized delivery of chemotherapeutic agents for cutaneous applications.^6,7^ By releasing drugs directly at the tumor site, these films can reduce systemic adverse effects and improve therapeutic outcomes.^6–9^ They may be composed of various natural and synthetic polymers, such as polyanhydrides, polycarbonates, polyesters, caprolactones, sodium alginate, hyaluronic acid, dextran, and chitosan.^10,11^

In parallel, the valorization of agro-industrial residues for the development of functional biomaterials has gained momentum in both scientific and industrial fields.^12,13^ In this context, watermelon rind (*Citrullus lanatus*, var. Crimson Sweet) has been identified as a promising source of bioactive compounds with therapeutic potential. Phenolic constituents, such as 4-hydroxybenzoic acid, quercetin, and coumaric acid, exhibit antioxidant and antimicrobial activities.^14–18^ Furthermore, El Gizawy et al.^19^ demonstrated that aqueous extracts of watermelon rind significantly inhibited cell proliferation, induced apoptosis, and suppressed cell migration in human cancer cell lines by modulating caspase-3 activity and BAX/BCL-2 expression. Similarly, Reddy et al.^20^ reported that watermelon rind extract reduced cell viability, altered gene expression profiles, and activated apoptosis pathways in human renal adenocarcinoma cells. They identified citrulline as a relevant metabolite that contributes to the extract’s bioactivity.

Our research group has been dedicated to the development of plant-based biopolymers, establishing methodologies for their extraction and preparation from agricultural waste products, such as onion bulbs^21–23^ and watermelon rind.^24,25^ The novelty of the present study lies in the incorporation of 5-fluorouracil (5-FU), a well-established chemotherapeutic agent, into watermelon rind-derived polymeric films, thereby combining the intrinsic bioactive properties of the rind with the pharmacological effects of 5-FU. This approach not only transforms underutilized agricultural byproducts into functional biomaterials but also advances sustainability by reducing the environmental impact and generating value-added products.

Therefore, the objective of this study was to optimize the production of watermelon rind-based films for 5-FU incorporation and to evaluate whether the therapeutic activity of the drug is retained, thus providing a sustainable system for localized skin cancer therapy.

## 2 Experimental Section

### 2.1 Preparation of Biopolymeric Films (WF)

Biopolymeric films derived from the white rind (mesocarp) of watermelons (WF) were prepared by using the solvent casting technique at BioSmart Nanotechnology. This biotechnology company is based in Araraquara, São Paulo, Brazil. The methodology was adapted from Dias et al.^21^ The process began with washing and manually separating the white rind portion of watermelons purchased from local markets in Araraquara (21°47′31″S, 48°10′52″W). The rind underwent hydrothermal treatment at 1.2 kgf/cm^2^ pressure for 5 min, yielding pulp. The pulp was then processed using an ultra-Turrax disperser (Ultra-380 Ultra Stirrer) at 7000 rpm for 5 min to obtain a purée. Prior to biopolymer preparation, the dry mass content was determined using a moisture analyzer (Marte ID200). The purée was then diluted in deionized water to a final concentration of 1% w/v. For the drug-loaded films (WF-5FU), 5-fluorouracil (5-FU, Sigma) was incorporated into the purée at a concentration of 100 μg/g relative to the total dry mass. The mixture was homogenized, and 30 g was poured into the Petri dishes (Kasvi, 80 × 15 mm^2^) and dried in a circulation-air incubator at 50 °C. Drug-free films (WF-Control) were prepared using the same methodology for comparative analyses.

### 2.2 Characterization of WF-5FU and WF-Control Biopolymers

#### 2.2.1 Scanning Electron Microscopy (SEM)

The morphological characteristics of the WF-5FU and WF-Control films were analyzed by using a JEOL JSM-IT500HR scanning electron microscope (Musashino, Tokyo, Japan). The films were fractured cryogenically in liquid nitrogen to obtain cross-sectional images. The analysis was performed under an accelerating voltage of 20 kV, utilizing the secondary electron detection mode. Prior to imaging, all samples were coated with a thin carbon layer to improve the conductivity and optimize the image resolution.

#### 2.2.2 Fourier Transform Infrared Spectroscopy (FTIR-ATR)

The functional groups of the WF-5FU and WF-Control biopolymeric films were evaluated by using FTIR with an attenuated total reflectance (ATR) configuration. Spectra were acquired using a Cary 630 spectrophotometer (Agilent Technologies, Santa Clara, CA), with a scanning range of 4000–400 cm^–1^ and 32 scans at a resolution of 4 cm^–1^.

#### 2.2.3 Thermal Behavior

Thermogravimetry (TG), derivative thermogravimetry (DTG), and differential scanning calorimetry (DSC) analysis curves were obtained using an SDT Q600 simultaneous TGA/DTG-DSC analyzer (TA Instruments, New Castle, DE). Measurements were conducted under a nitrogen atmosphere in the temperature range of 30–600 °C, with a sample mass of approximately 7.5 mg and a heating rate of 10 °C/min.

#### 2.2.4 Mechanical Properties

Mechanical properties were evaluated using a uniaxial tensile test following ASTM D882-18 standard.^26^ The analysis was performed using a TA.XT PLUS texture analyzer (Stable Micro Systems), equipped with a 50 kgf load cell. The test was conducted at a constant speed of 0.83 mm/s with an initial grip separation of 8 mm. Film specimens (100 × 25 mm^2^) were cut using an acrylic mold, and their dimensions were confirmed using a digital micrometer. Tensile strength, Young’s modulus, and elongation at break were determined from the recorded data. All tests were performed in quintuplicate at 25 °C and 50% relative humidity.

#### 2.2.5 Water Absorption and Swelling Rate

Film samples (2 cm^2^) were weighed initially to determine their dry mass. Then, they were individually immersed in 50 mL of deionized water at 25 ± 2 °C for 60 min to evaluate their water absorption capacity. After immersion, the samples were carefully removed and excess water was blotted with absorbent paper. The films were then reweighed to obtain their wet mass. The swelling ratio was calculated as the percentage increase in mass relative to the initial dry weight of the films.

#### 2.2.6 Barrier Properties

The water vapor transmission rate (WVTR) was determined by calculating the amount of water that permeated through the film over a given period and considering the exposed surface area available for vapor transmission.^27^ Based on this result, water vapor permeability (WVP) was calculated considering film thickness, relative humidity gradient, and vapor pressure difference across the film. Film thickness was measured by using an external digital micrometer (Shahe, 0–25 mm) with an accuracy of 0.001 mm. Measurements were taken at 10 random points on each film, and the mean value was recorded. Experiments were conducted using perforated flasks (Ø = 30 mm, height = 75 mm) containing 5 g of anhydrous calcium chloride, which was previously dried at 200 °C for 1 h. Circular film samples (15 mm in diameter) were positioned over the flask openings and placed inside a reactor chamber maintained at 25 ± 2 °C. The chamber contained 100 mL of a 23% w/w sodium chloride solution, creating an environment with a relative humidity of approximately 75% and a vapor pressure of 31.824 mmHg (equivalent to 4.242 kPa). To monitor moisture absorption, the weight of the flasks was recorded at hourly intervals over 8 h, and these data were used to determine the films’ permeability characteristics.

#### 2.2.7 Fluid Handling Capacity (FHC)

To determine FHC, films were positioned over the perforated lid of a modified Paddington cup system. The films were sealed with a ring and subjected to a controlled environment with pressure below 20 mmHg within a climate chamber set at 37 °C.^28^ First, the weight of the cup containing the film was recorded. Then, 20 mL of a saline solution was added to the cup. After 24 h, the cup was weighed again before and after removing the remaining fluid. These measurements allowed for a calculation of the amount of fluid absorbed and retained by the film. This provides insight into its fluid uptake and retention capabilities, which are key parameters for evaluating its potential performance in wound exudate management.^24^

### 2.3 Biological Assays

#### 2.3.1 Cell Lines and Culture Conditions

Murine melanoma (B16–F10, ATCC CRL-6475) and human keratinocyte (HaCaT, ATCC PCS-200-011) cell lines were obtained from the Rio de Janeiro Cell Bank (BCRJ). They are stored in an ultralow temperature freezer at −80 °C in the Cell Biobank of the Laboratory of Mutagenesis and Toxicity (LAMUT) at Uniara in Araraquara, São Paulo, Brazil. For experimental procedures, the cells were thawed and cultured in Dulbecco’s Modified Eagle Medium (DMEM, Sigma-Aldrich) supplemented with 10% fetal bovine serum (FBS, Gibco) in standard cell culture flasks (Kasvi). The cultures were maintained under controlled conditions at 37 °C in a humidified atmosphere with 5% CO_2_. Subculturing was performed by trypsinization as needed based on the confluence of each cell line.

#### 2.3.2 In Vitro Cytotoxicity Assessment—Resazurin-Based Cell Viability Assay

The cytotoxicity of the biopolymeric films (WF-5FU and WF-Control) was evaluated in HaCaT and B16–F10 cells using resazurin hydrochloride (Sigma-Aldrich) as an indicator of cell viability. Resazurin undergoes a colorimetric and fluorescence shift in response to cellular metabolic activity, serving as an indirect measure of cell viability. The assay was performed according to the protocol described by Page et al.^29^

Prior to testing, the films were sterilized with ultraviolet (UV) radiation for 1 h, and the eluates were prepared according to ISO 10993-12 ^30^ guidelines. For each film with a surface area of 6 cm^2^, 1 mL of DMEM supplemented with 10% FBS was added, and the samples were incubated at 37 °C under constant agitation for 24 h to allow for extraction.

For the assay, cells were seeded in 96-well microplates at a density of 1.0 × 10^4^ cells/well and incubated for 24 h to allow adhesion. Then, the cells were exposed to different concentrations of film eluates (1.6–50% v/v) for 48 h. After exposure, the culture medium was replaced with 100 μL of an aqueous resazurin solution (0.01% w/v), and the plates were incubated for an additional 4 h. Fluorescence was then measured using a Cytation microplate reader (BioTek) with excitation and emission wavelengths set at 530 and 590 nm, respectively. A 50% dimethyl sulfoxide (DMSO) solution served as the positive control for cytotoxicity, and untreated cells served as the negative control. All treatments were performed in triplicate and in three independent experiments. Cell viability was expressed as a percentage relative to the untreated control group, whose fluorescence intensity was considered 100%.

#### 2.3.3 Clonogenic Survival Assay

To evaluate the long-term effects of WF-5FU and WF-Control films on cell proliferation and survival, a clonogenic survival assay was performed based on the Franken et al.^31^ protocol, with modifications.

HaCaT and B16–F10 cells were seeded at a density of 1.0 × 10^5^ cells/well in 2 mL of complete culture medium in 6-well plates and incubated under standardized culture conditions (37 °C, 5% CO_2_, humidified atmosphere) for 24 h. The cells were then exposed to different concentrations (1.6–25% v/v) of biopolymeric film eluates prepared according to ISO 10993-12 ^30^ for 48 h. The experimental control consisted of untreated cells (negative control).

After treatment, the cells were washed, trypsinized, and counted using a Neubauer chamber. The cell concentration was adjusted to 200 cells/well, and the suspensions were reseeded in fresh six-well plates and incubated for an additional 7 days to allow colony formation. After incubation, the colonies were fixed with a methanol/acetic acid/distilled water solution (1:1:8) for 30 min and stained with a 2 mL/well Giemsa solution (Sigma-Aldrich) for 20 min. Three independent experiments were conducted. The survival fraction (SF%) was calculated by normalizing the number of colonies formed in each treated group to the number formed in the untreated control group and considered 100%.

#### 2.3.4 Cell Migration Assay

Cell migration was assessed both qualitatively and quantitatively using the methodology described by Martinotti and Ranzato.^32^ HaCaT and B16–F10 cells were seeded in 24-well plates at an initial density of 2 × 10^5^ cells/well. The plates were then incubated at 37  °C in a 5% CO_2_ atmosphere until approximately 90% confluence. To inhibit cell proliferation, the cultures were treated with mitomycin C (5 μg/mL) for 2 h. After this period, a uniform cell-free area was created in the confluent monolayer by scratching it with the tip of a sterile 200 μL pipet. Detached cells and debris were removed by washing with phosphate-buffered saline (PBS). Then, the cells were exposed to biopolymeric film eluates (WF-5FU and WF-Control) at a concentration of 6.2% v/v. The control group received complete culture medium without treatment (negative control). Cell migration into the cell-free area was monitored using a digital camera (DFC7000T) coupled to an inverted microscope (Leica DMi8, Frankfurt, Germany) and quantified using ImageJ software (NIH, Bethesda, MD). Cell migration percentage was calculated by comparing the cell-free region immediately after the scratch (At = 0 h) with the region measured after 24 h (At = 24 h). Results were expressed as the mean of three independent experiments.

#### 2.3.5 Mutagenicity Assessment—Micronucleus (MN) Assay

The micronucleus (MN) assay was conducted according to Fenech,^33^ with adaptations. HaCaT and B16–F10 cells were exposed to eluates from the biopolymeric films WF-Control (3.1–25% v/v) and WF-5FU (1.6–25% v/v) for 48 h. The eluates were prepared by incubating each film (6 cm^2^) with 1 mL of culture medium for 72 h at 37  °C under constant agitation, following ISSO 10993-12.^30^

The cells were seeded in six-well plates at a density of 1 × 10^5^ cells/well and incubated for 24 h to allow for adhesion. Complete culture medium served as the negative control, and hydrogen peroxide (100 μM) served as the positive control. To block cytokinesis, cytochalasin B (3.0 μg/mL) was added for a duration equivalent to one and a half cell cycles. The cells were then trypsinized, centrifuged, subjected to hypotonic treatment with potassium chloride (0.075 M KCl), and fixed in methanol/acetic acid (3:1). Cell suspensions were dropped onto clean slides, air-dried, stained with Giemsa, and examined under a light microscope at 40× magnification. The frequency of micronuclei was evaluated in a total of 3000 viable binucleated cells per treatment group (1000 per replicate), following Fenech’s^33^ criteria. Additionally, 500 viable cells (including mononucleated, binucleated, and multinucleated cells) per replicate were counted to determine the cytokinesis-block proliferation index (CBPI), as described by OECD,^34^ to assess cellular toxicity and cell cycle delay.

### 2.4 Statistical Analysis

Analyses were performed using Student’s *t*-test for physicochemical parameters (thickness, mechanical properties, and swelling rate), with statistical significance defined at *p* < 0.05. Biological assays were analyzed using GraphPad Prism 7.0 software. One-way analysis of variance (ANOVA) followed by a Tukey post hoc test was applied to multiple comparisons with a significance level set at *p* < 0.05. The results were interpreted based on comparisons with the negative control.

## 3 Results and Discussion

### 3.1 Characterization of WF-5FU and WF-Control Biopolymers

The WF-Control and WF-5FU biopolymeric films were produced using the casting method and an ecofriendly strategy that did not involve the addition of plasticizers or supplementary biopolymers. This approach resulted in uniform, crack-free surfaces. The casting process involves dissolving the polymer in an appropriate solvent and depositing the resulting filmogenic solution in a mold. Controlled solvent evaporation then leads to film formation.^10^

Fruit purées derived from the watermelon rind, obtained through hydrothermal treatment, is rich in pectic and cellulosic components that confer film-forming capabilities. In these matrices, naturally occurring sugars act as plasticizers, and the high fiber content contributes to improved mechanical strength and thermal stability.^35^ During the hydrothermal process, pectin functions as a natural binding agent, which enhances the cohesion and structural integrity of the resulting films.^24^ Furthermore, pectin can form a stable matrix with cellulose and hemicellulose, which reinforces the film’s architecture.^21,36^

Macroscopically, incorporating 5-FU into the biopolymer matrix did not produce noticeable alterations in the films’ visual or morphological characteristics, as shown in Figure 1A1–B1. Both WF-Control and WF-5FU presented a brownish coloration, attributed to compounds formed via Maillard reactions during hydrothermal processing. The films also exhibited homogeneous surfaces and notable transparency, consistent with the findings of Paris Junior et al.^25^

**Figure 1:**
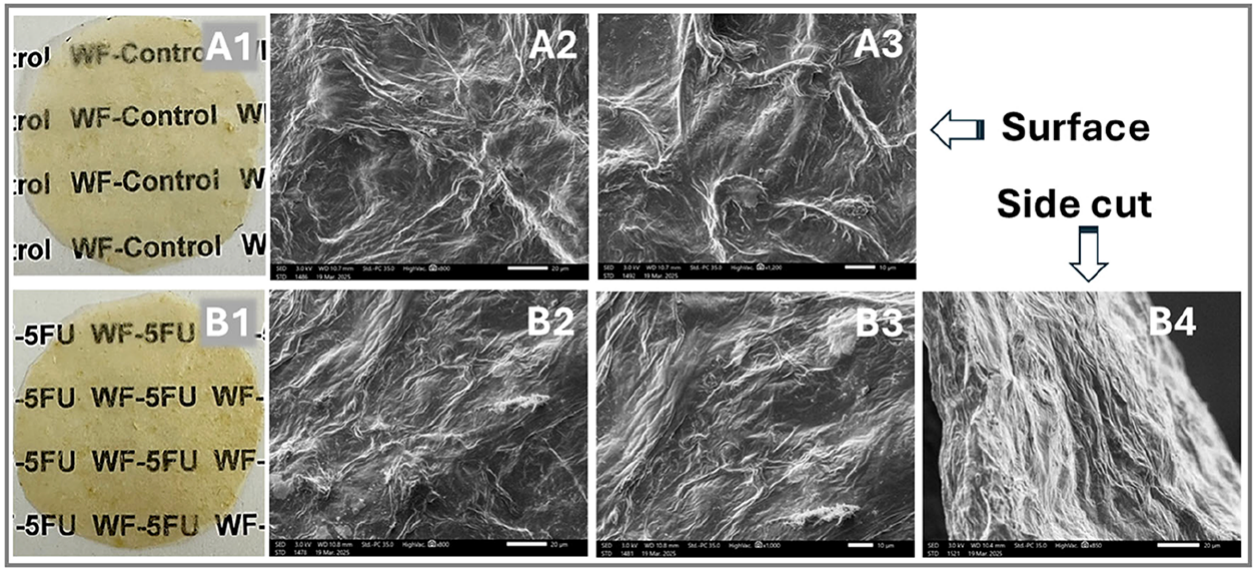
Film photographs of (A1) WF-Control and (B1) WF-5FU. SEM micrographs of the surface for WF-Control (magnification: 800× [A2] and 1200× [A3]) and WF-5FU (magnification: 800× [B2] and 1000× [B3]), as well as a side cut (magnification: 850× [B4]).

SEM analysis of the WF-Control film revealed a moderately rough surface with undulations and dispersed fibers throughout the structure (Figure 1A2,A3). Similarly, the SEM images of WF-5FU (Figure 1B2,B3) showed comparable morphological features, maintaining the surface roughness and heterogeneity observed in the control film. These results suggest that incorporating 5-FU did not significantly impact the film’s microstructure. Additionally, the cross-sectional SEM image of the WF-5FU film (Figure 1B4) revealed an irregular, multiplanar profile.

Figure 2A1,A2 shows the FTIR spectra of the WF-Control film. Figure 2A1 shows a broad absorption band between 3600 and 2990 cm^–1^, with a maximum at 3296 cm^–1^. This band is mainly attributed to O–H stretching vibrations from hydroxyl groups, which are characteristic of pectin, lignin, and cellulose—the key structural components of watermelon white rind.^24,25,37^ In pectin, this band arises from the O–H stretching of hydroxyl and carboxylic acid groups, influenced by hydrogen bonding and hydration.

**Figure 2:**
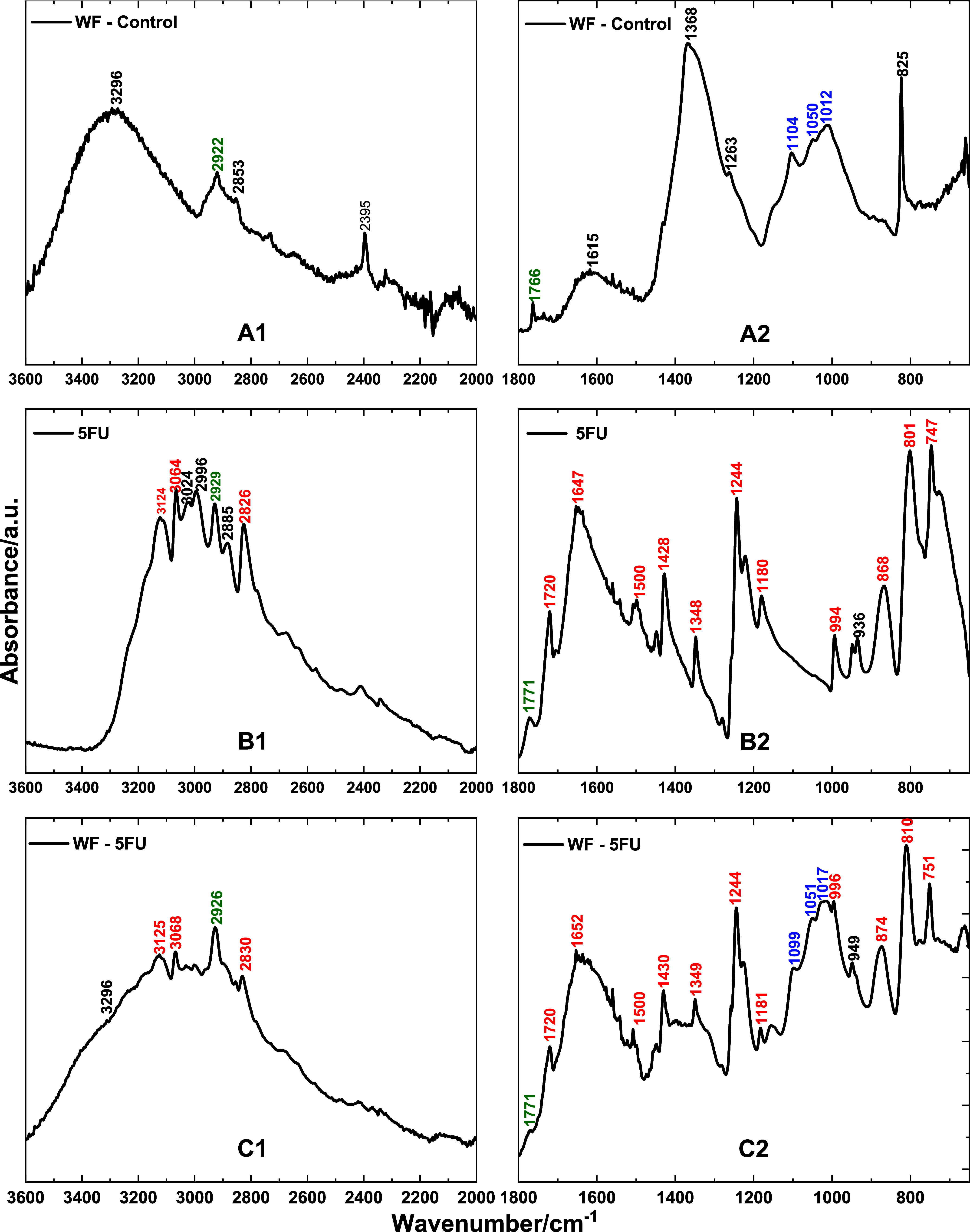
Infrared spectra of a biopolymeric watermelon film (WF-Control, A1, A2), pure 5-fluorouracil drug (5-FU, B1, B2), and 5-FU-incorporated films (WF-5FU, C1, C2). (1) Upper region: 4000–2000 cm^–1^; (2) Lower region: 2000–400 cm^–1^.

Figure 2A2 shows the additional bands. The distinct band at approximately 1733 cm^–1^ corresponds to the C=O stretching of esterified carboxyl groups in pectin. A broader, less intense band at 1615 cm^–1^ is associated with the C=O stretching of carbonyl groups. The strong and broad absorption around 1368 cm^–1^ likely arises from C–H bending or C–O stretching, mainly from cellulose and pectin. The band at 1263 cm^–1^ corresponds to C–O–C stretching in glycosidic linkages and ester groups. Finally, the region between 1050 and 1012 cm^–1^ is characteristic of cellulose and represents C–O stretching from the primary and secondary alcohols of glucose units.^37^

Figure 2B1,B2 displays the FTIR spectrum of pure 5-FU, and Figure 2C1,C2 shows the spectrum of the WF-5FU film. The characteristic absorption bands of 5-FU are clearly identifiable in the WF-5FU spectrum and are highlighted in red. The band at 3124 cm^–1^ is attributed to N–H stretching in the pyrimidine ring. The band at 3164 cm^–1^ corresponds to aromatic C–H stretching vibrations. The band near 1720 cm^–1^ represents C=O stretching of the carbonyl group, while the signal at 1647 cm^–1^ also reflects C=O stretching in the pyrimidine ring. The absorption at 1428 cm^–1^ is likely associated with C–H or N–H bending vibrations. The 1244 cm^–1^ band is characteristic of C–F stretching, and the bands at 868 and 747 cm^–1^ correspond to out-of-plane C–H bending in the fluorinated pyrimidine ring. These signals confirm the presence of 5-FU in the biopolymeric matrix, likely through physical entrapment or solubilization without significant chemical interaction.

Figure 3 shows the TG/DTG-DSC curves of the WF-Control and WF-5FU films. Table 1 summarizes the thermal events obtained from the deconvolution of the DTG curves using the bi-Gaussian function in OriginPro (Version 2025, OriginLab Corporation, Northampton, MA). The table reports the peak temperature (*T*__peak_), the onset and end temperatures, and the corresponding mass loss (%), as determined by TG analysis.

**Figure 3:**
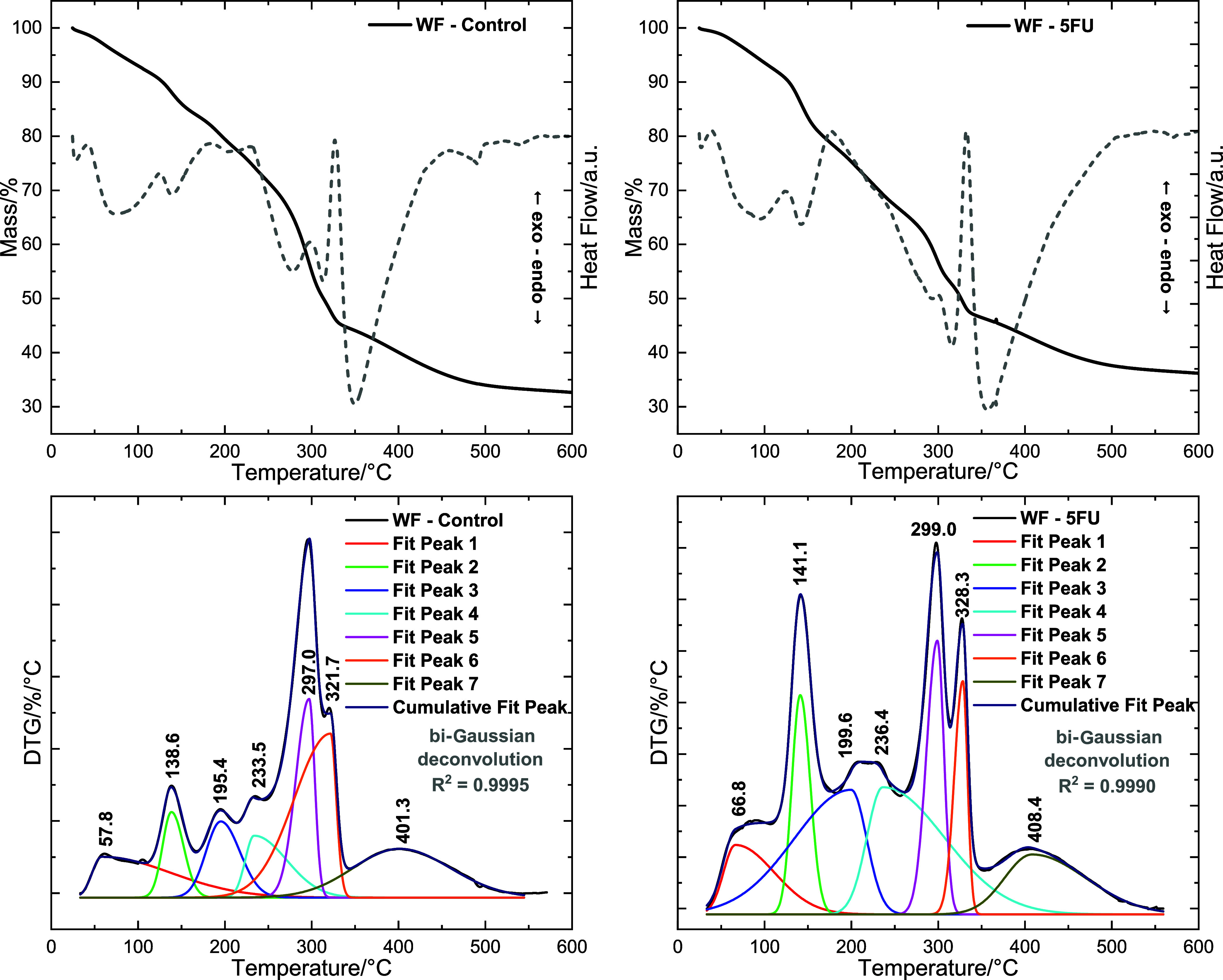
TG/DTG and DSC analyses of the biopolymeric watermelon film (WF-Control) and 5-fluorouracil-incorporated film (WF-5FU). Deconvolution of DTG curves was performed using the bi-Gaussian function (OriginLab 2025).

**Table 1:** Summary of Thermal Decomposition Data Obtained from TG/DTG Analysis of the WF-Control and WF-5FU Films, Including Deconvoluted DTG Peak Temperatures (*T*__peak_), Onset and End Temperatures, and Corresponding Mass Losses for Each Thermal Event

fit peak	*T*__peak_/°C	*T*_ini_/°C	*T*_end_/°C	mass loss/%
WF-Control				
P1	57.8	33.4	362.1	6.93
P2	138.6	93.1	197.7	4.62
P3	195.4	118.7	292.1	6.42
P4	233.5	184.6	372.9	6.27
P5	297.0	234.1	332.5	10.78
P6	321.7	149.9	353.5	19.66
P7	401.3	202.3	544.6	11.51
residue at 544.6 °C		33.15%	total	66.19

WF-5FU				
P1	66.8	33.4	232.3	5.05
P2	141.1	101.3	189.6	6.80
P3	199.6	33.4	269.8	14.93
P4	236.4	166.2	494.4	15.90
P5	299.0	247.0	332.4	8.16
P6	328.3	284.4	355.2	5.16
P7	408.4	284.4	559.4	6.94
residue at 559.4 °C		36.63%	total	62.94

For the WF-Control film, the first three DTG peaks represent moisture loss and dehydration, followed by the volatilization and decomposition of low-molecular-weight compounds,^37^ such as residual sugars and organic acids that are formed during biopolymer production. Peaks 4 to 7 correspond to the degradation of the pectin–lignocellulosic matrix, accounting for ∼48.22% of the total mass loss. A residual carbonaceous fraction of 33.15% remained at 544.6 °C (Table 1).

The WF-5FU film exhibited a thermal profile similar to that of the WF-Control. Typically, 5-FU shows a melting peak, followed by decomposition near 275 °C.^38^ The absence of this transition confirms that the drug is not present in the crystalline form.

DSC analysis corroborates these results (Figure 3). Both the WF-Control and WF-5FU films displayed only endothermic transitions that matched the mass loss events detected in the TG curves.

The incorporation of 5-FU resulted in a slight increase in film thickness. However, this difference was not statistically significant, likely because the dry matter content was consistent across the formulations. WF-Control measured 0.122 ± 0.02 mm, and WF-5FU measured 0.144 ± 0.02 mm (Table 2). Film thickness is a critical parameter because it directly influences mechanical strength, gas and moisture barrier properties, transparency, flexibility, and durability.^39^ Therefore, specific measures were implemented during film production to ensure uniformity and optimize their structural and functional characteristics for potential biomedical applications.

**Table 2:** Thickness, Mechanical Properties, Swelling Rate (SR %), Barrier Properties, and Fluid Handling Capacity of the Biopolymeric Watermelon Film (WF-Control) and 5-Fluorouracil-Incorporated Film (WF-5FU)^a^

	WF-Control	WF-5FU
thickness		
mm	0.122 ± 0.02	0.144 ± 0.02

mechanical properties		
σ*T* (MPa)	0.23 ± 0.03	0.36 ± 0.02^b^
*E* (MPa)	136.7 ± 27.0	134.7 ± 18.1
ε*R* (%)	1.5 ± 0.10	2.5 ± 0.17^b^

swelling rate (SR %)		
SR (%)	1422 ± 60.8	2170 ± 72.4^b^

barrier properties		
α (g·h^–1^)	0.45	1.55
WVTR (g·h^–1^·m^–2^)	1303.9	4471.8
WVP (g·mm·m^–2^·day^–1^·mmHg^–1^)	3.7	15.2

fluid handling capacity (g·m^–2^·day^–1^)		
MVTR	215.8	267.6
ABS	10.8	37.2
FHC	226.6	304.7

a α: permeability coefficient; WVTR: water vapor transmission rate; WVP: water vapor permeability; MVTR: moisture vapor transmission rate; ABS: absorbency; FHC: fluid handling capacity.

b Statistically different from WF-Control (*p* < 0.05, Student’s *t*-test, applied to thickness, mechanical properties, and swelling rate).

Due to the importance of mechanical performance in biomedical materials, the mechanical properties of WF-5FU were evaluated and compared to those of WF-Control. The analyzed parameters included tensile strength (σ*T*, MPa), Young’s modulus (*E*, MPa), and elongation at break (ε*R*, %); these are summarized in Table 2. These parameters collectively characterize the films’ mechanical behavior and structural integrity under tensile loading.

Tensile tests were conducted by applying a uniaxial load at a constant speed until rupture, which allowed for the evaluation of the key mechanical parameters. Tensile strength represents the maximum stress that the material can endure before failing, which corresponds to the onset of an irreversible deformation of the polymer chains. Young’s modulus, defined as the ratio of stress to strain within the elastic region, reflects the material’s stiffness. Elongation at break indicates the film’s ability to undergo deformation prior to rupture and serves as a measure of its flexibility and extensibility.^40^

The WF-Control sample exhibited the lowest tensile strength (0.23 ± 0.03 MPa) and elongation at break (1.5 ± 0.10%), indicating limited toughness and flexibility. In contrast, WF-5FU exhibited increased tensile strength (0.36 ± 0.02 MPa) and elongation at the break (2.5 ± 0.17%), suggesting greater extensibility and mechanical resistance. The Young’s modulus of WF-5FU (134.7 ± 18.1 MPa) was slightly lower than that of WF-Control (136.7 ± 27.0 MPa), which confirms a reduction in stiffness (Table 2). These changes may be attributed to weak intermolecular interactions, such as hydrogen bonding, between 5-FU and the biopolymeric matrix, which may have contributed to mechanical reinforcement of the film.

In addition to mechanical performance, the films’ water absorption capacity, barrier properties, and fluid handling ability were also evaluated. These are critical parameters for selecting effective wound dressings.^41^ Ideally, a wound dressing would have balanced permeability to maintain a moist wound environment while regulating drug release.^42^ In advanced stages of skin cancer, ulcerative lesions are often accompanied by excessive exudate due to disruption of the skin barrier, inflammation, and possible microbial infection. Therefore, a dressing’s ability to absorb and manage wound fluids is vital to promoting healing and improving patient comfort.^2,43^

The biopolymeric films demonstrated high swelling capacity with values of 2170 ± 72.4% for WF-5FU and 1422 ± 60.8% for WF-Control (Table 2). Moreover, WF-Control exhibited the lowest water vapor permeability (WVP = 3.7 g·mm·m^–2^·day^–1^·mmHg^–1^), indicating a superior barrier function, whereas WF-5FU showed the highest water vapor transmission rate (WVTR = 4471.8 g·h^–1^·m^–2^), permeability coefficient (α = 1.55 g·h^–1^), and fluid handling capacity (FHC = 304.7, Table 2). Permeability, defined as the steady-state flow of a solute through a material, is closely related to water vapor transport driven by humidity gradients^21^ and directly influences both fluid management and drug release.

Taken together, these results suggest that 5-FU incorporation increased the film’s hydrophilicity, enhancing water uptake and molecular diffusion within the biopolymeric matrix. This effect is particularly relevant for managing exudative lesions and indicates that WF-5FU films may be suitable for topical application, favoring lesion coverage and maintenance of a hydrated microenvironment to support sustained 5-FU release.

### 3.2 Biological Assays

The in vitro cytotoxicity of the WF-Control and WF-5FU biopolymeric films was evaluated by using a resazurin reduction assay with HaCaT and B16–F10 cells. The WF-Control films exhibited no cytotoxic effects in either cell line, which confirms the biocompatibility of the polymeric matrix. In contrast, the WF-5FU films induced a statistically significant and concentration-dependent reduction in cell viability compared to the negative control (untreated cells), which indicates the effective release of 5-FU from the films.

In HaCaT cells, the WF-5FU film was cytotoxic at an eluate concentration of 12.5% v/v, reducing the cell viability to 62.3 ± 13.0%. This effect intensified with increasing concentrations, reaching 47.5 ± 9.7% at 25% v/v and 30.1 ± 4.3% at 50% v/v. In B16–F10 cells, the cytotoxic response was more pronounced; the viability decreased from 79.2 ± 16.4% at 1.56% v/v to 8.2 ± 1.2% at 50% v/v. Statistically significant reductions were observed at all tested concentrations (Figure 4). These results confirm the antitumor activity of 5-FU released from the films and suggest that B16–F10 cells are more sensitive to treatment than HaCaT cells, likely due to differences in metabolic rate and proliferative behavior.

**Figure 4:**
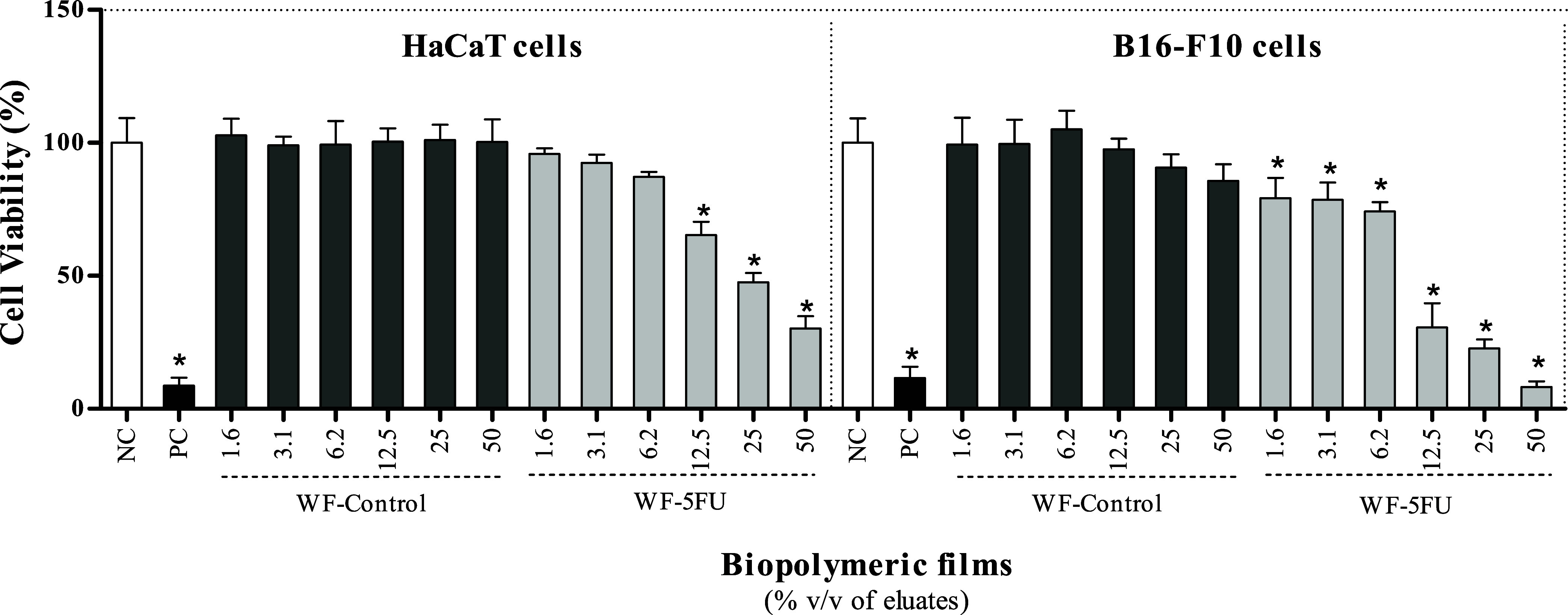
Cytotoxicity assays using resazurin as an indicator of cell viability in HaCaT and B16–F10 cells treated with different concentrations of eluates of the biopolymeric watermelon white rind film (WF-Control) and 5-fluorouracil-incorporated film (WF-5FU). NC: negative control (DMEM supplemented with 10% fetal bovine serum), cell viability of 100%; PC: positive control (dimethyl sulfoxide, DMSO, 50%), cell viability of 8.6 ± 2.9% (HaCaT) and 11.5 ± 4.2% (B16–F10). Values are expressed as the mean ± standard deviation of replicates from three independent assays. *Statistically different from the NC (*p* < 0.05, one-way ANOVA followed by Tukey’s post hoc test).

To further investigate the long-term effects of treatment on cell proliferation, we performed a clonogenic survival assay was performed. This assay is commonly used in tumor biology to evaluate the capacity of individual cells to survive treatment and develop into colonies. Only cells with clonogenic potential can contribute to tumor recurrence or metastasis.^49^

WF-5FU eluates significantly reduced the clonogenic survival in both HaCaT and B16–F10 cells in a concentration-dependent manner (Figure 5A). These results corroborate those of the resazurin assay and demonstrate both cytostatic and cytotoxic activity. These findings suggest that WF-5FU films compromise cellular metabolic activity and impair the long-term proliferative potential of the treated cells.

**Figure 5:**
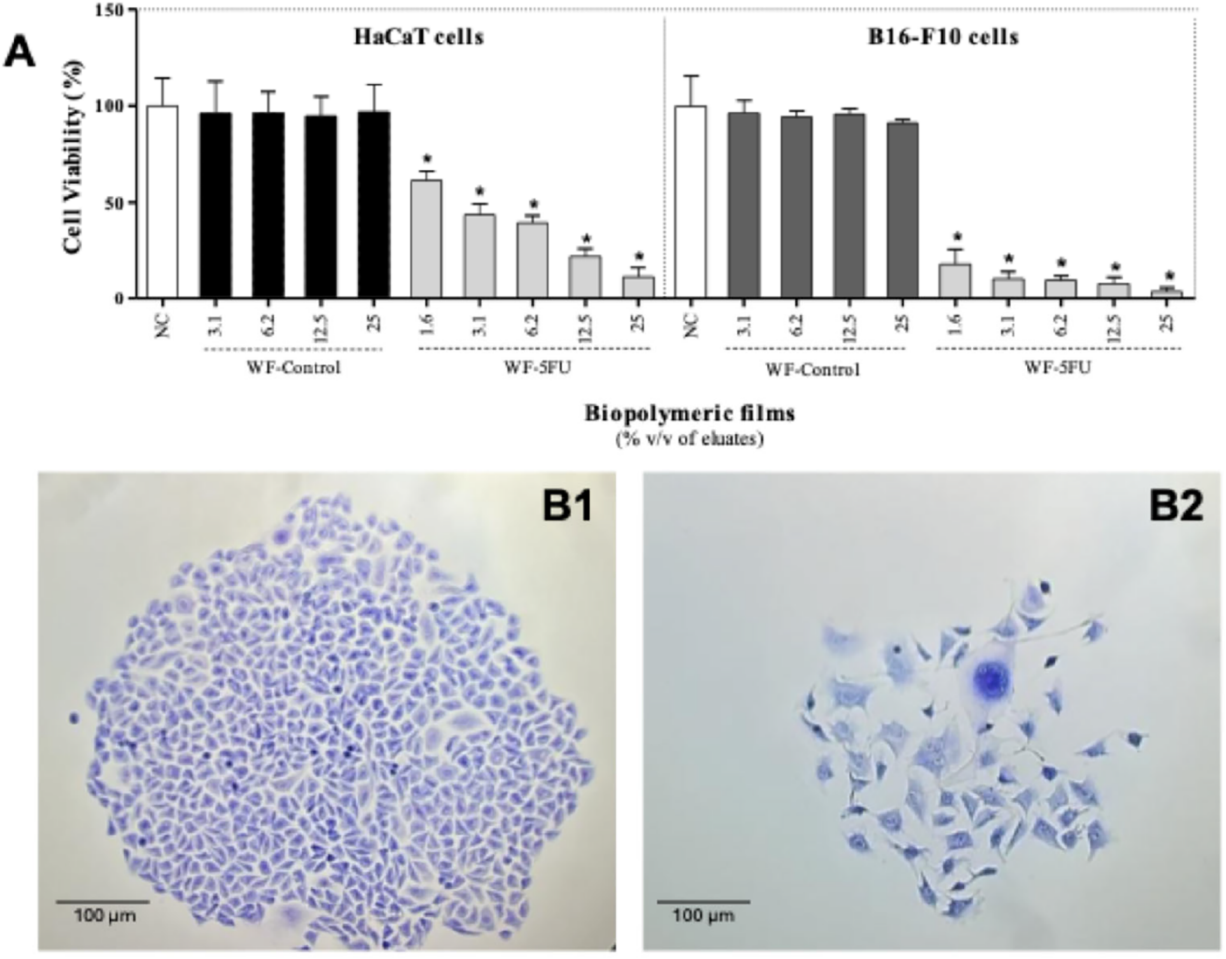
(A) Survival fraction (%) of HaCaT and B16–F10 cells treated with eluates of the biopolymeric watermelon white rind film (WF-Control) and 5-fluorouracil-incorporated film (WF-5FU). (B) Representative images of B16–F10 cell colonies (scale bar: 100 μm): (B1) colonies in the wells corresponding to the negative control and (B2) colonies after treatment with WF-5FU films (25% v/v eluate). NC: negative control (DMEM supplemented with 10% fetal bovine serum), survival fraction of 100%. Values are expressed as the mean ± standard deviation of replicates from three independent assays. *Statistically different from NC (*p* < 0.05, one-way ANOVA followed by Tukey’s post hoc test).

Visual inspection of the clonogenic assay wells (Figure 5B1,B2) corroborated these observations, revealing a significant decrease in colony number and size in the WF-5FU-treated groups (Figure 5B2) compared to the negative control (Figure 5B1), accompanied by evident cellular and nuclear morphological alterations in B16–F10 cells.

The observed cytotoxic effects are consistent with the well-established mechanism of action of 5-FU, a chemotherapeutic antimetabolite widely used in the treatment of skin cancers.^44–46^

5-FU primarily inhibits thymidylate synthase (TS) and incorporates its active metabolites into DNA and RNA. Following administration, most 5-FU is metabolized in the liver to its inactive form, dihydrofluorouracil, while a fraction is converted into active metabolites, including fluorodeoxyuridine monophosphate (FdUMP), fluorodeoxyuridine triphosphate (FdUTP), and fluorouridine triphosphate (FUTP). FdUMP inhibits TS, which blocks the production of deoxythymidine monophosphate (dTTP), which is an essential nucleotide for DNA synthesis and repair. This leads to DNA strand breaks. Additionally, the incorporation of FUTP into RNA impairs its processing and function, further contributing to cytotoxicity.^47^

Due to the pharmacokinetic limitations of 5-FU, including its short plasma half-life (5–20 min) and rapid hepatic metabolism, high systemic doses are often required to achieve therapeutic levels, frequently resulting in adverse effects on healthy, proliferating tissues.^48,49^ Thus, the biopolymeric films based on the watermelon rind developed in this study offer a promising platform in skin cancer therapy. Concentrating the drug at the tumor site may maintain effective local concentrations, reduce dosing frequency, and minimize off-target toxicity.^50^

Additionally, the migratory capacity of HaCaT and B16–F10 cells was quantitatively evaluated by measuring the reduction in the cell-free area in the monolayer (Figure 6A) and qualitatively assessed it through photomicrographs captured 24 h after treatment (Figure 6B), which illustrated the extent of cell displacement.

**Figure 6:**
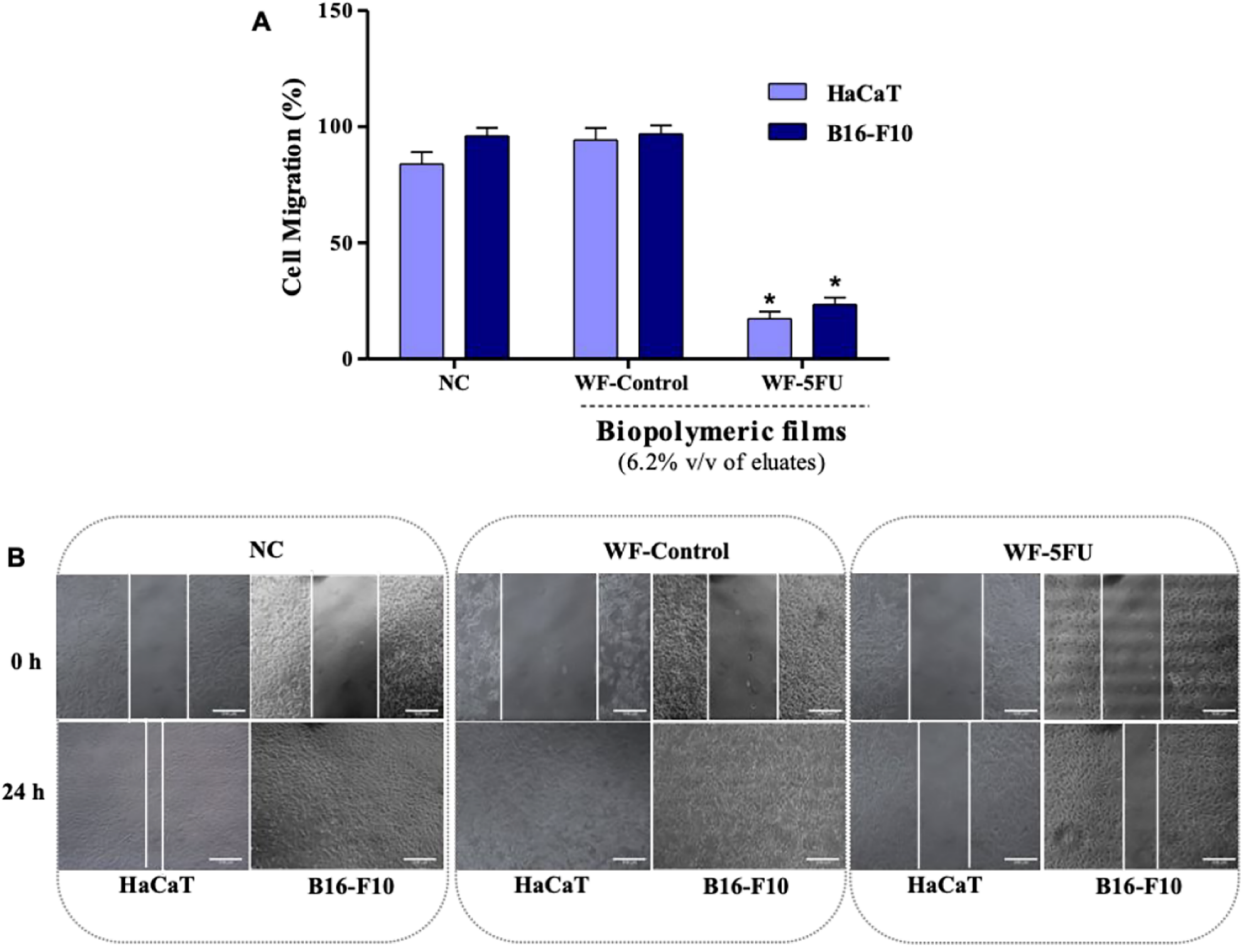
(A) Quantitative analysis of the cell migration area of HaCaT and B16–F10 cells after treatment with eluates (6.2% v/v) of the biopolymeric watermelon white rind film (WF-Control) and the 5-fluorouracil-incorporated film (WF-5FU). (B) Representative images of HaCaT and B16–F10 cells subjected to an in vitro scratch assay (scale bar: 200 μm). Images were captured immediately after scratch induction (0 h) and after 24 h of incubation. NC: negative control (DMEM supplemented with 10% fetal bovine serum). Results represent three independent experiments. *Indicates an inhibitory effect, and values are statistically different from the NC (*p* < 0.05, one-way ANOVA followed by Tukey’s post hoc test).

Cell migration plays a pivotal role in tumor progression and metastasis. In epithelial-derived carcinomas, metastatic spread involves a series of steps, including loss of epithelial polarity, disruption of tissue architecture, degradation of the basement membrane, intravasation into the circulatory or lymphatic systems, and colonization of distant tissues.^51^ Thus, limiting migratory capacity can significantly impair the invasive and metastatic potential of tumor cells.^52^

Shenoy et al.^53^ demonstrated that both free 5-FU and its solid lipid nanoparticle formulation significantly inhibited B16–F10 cell migration, even at subtoxic concentrations (0.12 μg/mL), compared to the untreated control.

In this study, WF-Control films did not alter cell migration in either cell line, and no statistically significant differences were observed compared to the negative control. In contrast, WF-5FU films significantly inhibited cell migration in both cell lines. The percentage reduction in the cell-free area was 17.3 ± 3.0% for HaCaT cells and 23.4 ± 3.1% for B16–F10 cells (Figure 6A). The antimigratory effect of WF-5FU films further underscores their clinical relevance as it may contribute to reducing the risk of metastasis.

The micronucleus (MN) assay was used to evaluate genomic instability in HaCaT and B16–F10 cells following exposure to eluates from biopolymeric films. Additionally, nuclearity analysis was used to determine the cytokinesis-block proliferation index (CBPI), a key parameter for evaluating cellular toxicity and cell cycle delay.^34^ This assay is widely used in genetic toxicology to assess the safety of chemicals, pharmaceuticals, and cosmetics for human use. MN arise from chromosome fragments or entire chromosomes that fail to be incorporated into the main nucleus after cell division and are established biomarkers of DNA damage.^54^

The WF-Control films did not induce mutagenic effects in either cell line, which reinforces the biocompatibility of the biopolymer and supports its potential for biomedical applications. As expected, the WF-5FU films produced a concentration-dependent increase in MN frequency (Table 3), consistent with the genotoxic effects of 5-FU.^55–57^ Mutagenicity was observed at eluate concentrations of 12.5 and 6.2% v/v. These concentrations also led to a significant decrease in CBPI (Table 3), indicating concomitant cytostatic or cytotoxic activity. This finding is supported by the resazurin and clonogenic assay data.

**Table 3:** Frequency of Micronuclei (MN) and Cytokinesis-Block Proliferation Index (CBPI) in HaCaT and B16–F10 Cells after 48 h of Treatment with Eluates of the Biopolymeric Watermelon White Rind Film (WF-Control) and 5-Fluorouracil-Incorporated Film (WF-5FU)^a^

	HaCaT		B16–F10	
treatments	MN^b^	CBPI^c^	MN^b^	CBPI^c^
NC	5.0 ± 1.5	1.8 ± 0.03	5.0 ± 1.7	1.8 ± 0.05
PC	28.5 ± 3.5^d^	1.6 ± 0.01^d^	36.0 ± 2.8^d^	1.5 ± 0.01^d^
WF-Control				
3.1%	5.5 ± 0.7	1.7 ± 0.06	4.5 ± 0.7	1.8 ± 0.03
6.2%	4.5 ± 0.7	1.7 ± 0.05	5.5 ± 2.8	1.7 ± 0.05
12.5%	5.0 ± 1.4	1.8 ± 0.08	5.0 ± 0.7	1.7 ± 0.01
25%	4.0 ± 1.4	1.7 ± 0.06	6.0 ± 1.4	1.8 ± 0.02
WF-5FU				
1.6%	5.5 ± 0.7	1.8 ± 0.04	6.5 ± 0.7	1.7 ± 0.06
3.1%	8.0 ± 1.4	1.7 ± 0.05	8.5 ± 2.1	1.7 ± 0.01
6.2%	12.5 ± 0.7^d^	1.6 ± 0.06^d^	14.0 ± 1.4^d^	1.6 ± 0.02^d^
12.5%	16.5 ± 2.1^d^	1.4 ± 0.02^d^	18.0 ± 2.8^d^	1.5 ± 0.01^d^

a Values are presented as mean ± standard deviation of the frequency of micronuclei (MN) and CBPI in HaCaT and B16–F10 cells. NC: negative control (DMEM supplemented with 10% fetal bovine serum), PC: positive control (hydrogen peroxide, 100 μM).

b A total of 3000 binucleated cells were analyzed per treatment group (1000 cells/treatment/repetition).

c A total of 1500 cells were analyzed per treatment group (500 cells/treatment/repetition).

d Statistically different from NC (*p* < 0.05, one-way ANOVA followed by Tukey’s post hoc test).

This response aligns with the established mechanism of action of 5-FU (a nucleoside analog), which was previously discussed and involves interference with DNA synthesis and integrity.^47,57^ While these mechanisms contribute to its antitumor efficacy, they may also lead to long-term genomic instability.^58^ However, this risk is minimized when drug release is restricted to the lesion site, representing a strategic advantage of topical therapeutic approaches.

## 4 Conclusions

This study contributes to our research group’s ongoing efforts to develop sustainable biopolymeric films from agro-industrial residues, aiming to create environmentally responsible platforms for biomedical applications. Watermelon white rind, an abundant and underutilized byproduct, was successfully repurposed to produce biopolymeric films incorporating 5-FU, a widely used chemotherapeutic agent.

The resulting films exhibited favorable physicochemical and mechanical properties, and in their drug-free form (WF-Control), they showed no cytotoxic or genotoxic effects, which reinforces the safety of the biopolymeric matrix. When loaded with 5-FU, the films demonstrated potent cytotoxic and antimigratory effects in vitro against HaCaT and B16–F10 cells. Furthermore, the WF-5FU eluates induced a concentration-dependent increase in MN frequency, which is consistent with the known mechanism of action of 5-FU.

These results suggest that watermelon rind-based biopolymeric films have potential as localized drug delivery systems for skin cancer therapy, especially for early-stage tumors or as adjuncts in combination regimens. By enabling localized administration, these films could reduce the systemic toxicity commonly associated with conventional 5-FU treatments, thereby strengthening their translational relevance.

Further research is needed, including in vivo studies and broader preclinical evaluations, to validate the therapeutic effectiveness of these sustainable biopolymeric platforms and to define their most suitable clinical applications.

## Data Availability

Data will be made available on request.
